# Linear Interval Approximation for Smart Sensors and IoT Devices

**DOI:** 10.3390/s22030949

**Published:** 2022-01-26

**Authors:** Marin B. Marinov, Nikolay Nikolov, Slav Dimitrov, Todor Todorov, Yana Stoyanova, Georgi T. Nikolov

**Affiliations:** 1 Faculty of Electronic Engineering and Technologies, Technical University of Sofia, 1756 Sofia, Bulgaria; gnikolov@tu-sofia.bg; 2 Faculty of Industrial Technology, Technical University of Sofia, 1756 Sofia, Bulgaria; nickn@tu-sofia.bg (N.N.); slav.dimitrov@gmx.net (S.D.); tst@tu-sofia.bg (T.T.); yast@tu-sofia.bg (Y.S.)

**Keywords:** approximation, IoT, linearization techniques, piecewise approximation, recourse constrained devices, smart sensors

## Abstract

In this work, we introduce and use an innovative approach for adaptive piecewise linear interval approximation of sensor characteristics, which are differentiable functions. The aim is to obtain a discreet type of inverse sensor characteristic, with a predefined maximum approximation error, with minimization of the number of points defining the characteristic, which in turn is related to the possibilities for using microcontrollers with limited energy and memory resources. In this context, the results from the study indicate that to overcome the problems arising from the resource constraints of smart devices, appropriate “lightweight” algorithms are needed that allow efficient connectivity and intelligent management of the measurement processes. The method has two benefits: first, low-cost microcontrollers could be used for hardware implementation of the industrial sensor devices; second, the optimal subdivision of the measurement range reduces the space in the memory of the microcontroller necessary for storage of the parameters of the linearized characteristic. Although the discussed computational examples are aimed at building adaptive approximations for temperature sensors, the algorithm can easily be extended to many other sensor types and can improve the performance of resource-constrained devices. For prescribed maximum approximation error, the inverse sensor characteristic is found directly in the linearized form. Further advantages of the proposed approach are: (i) the maximum error under linearization of the inverse sensor characteristic at all intervals, except in the general case of the last one, is the same; (ii) the approach allows non-uniform distribution of maximum approximation error, i.e., different maximum approximation errors could be assigned to particular intervals; (iii) the approach allows the application to the general type of differentiable sensor characteristics with piecewise concave/convex properties.

## 1. Introduction

### 1.1. Resource-Constrained Smart Sensor Devices—Definitions

In recent years, there has been a strong increase in the interest of scientists in smart, energy-saving sensor technologies. Many promising developments have been made in both sensor production and wireless communications. The implementation of complex sensor systems is already possible due to the high degree of miniaturization of the series of classical measurement methods. Research in the field of smart sensors and the Internet of Things (IoT) has been growing. Unfortunately, there is still a lack of clarity in the terminology used in the specialized literature [1]. 

#### 1.1.1. Smart Sensors

It is often considered that the term ‘smart sensors’ was created along with the term IoT. However, IEEE introduced a definition of smart transducers (sensors and actuators) in 1997 in its IEEE 1451 standard [2,3]. For transducers to be smart, IEEE 1451 requires them to be supplemented with specific hardware components (memory/processor) and additional functionality. The Transducer Electronic Data Sheet (TEDS) stored in the memory, contains data related to identification, calibration, and correction, as well as manufacturing information. 

#### 1.1.2. IoT Devices

The emergence of IoT and the advances in the field of microcontrollers and the Internet allowed the industry to build smart devices integrating complex sensor and communication functions as well as signal processing algorithms.

The definitions of xconventional sensors, smart sensors according to the IEEE 1451 standard, and smart IoT sensors can be summarized as follows.

According to NIST, a sensor is a device that transforms physical, chemical, and biological parameters into electrical signals and converts them into analog and/or digital data. A smart sensor, according to the IEEE 1451 standard, adds functionality to the NIST definition: the device can store data for sensor identification, calibration, correction, manufacturer-related information, and serves as a communication interface or processor. 

Additionally, at the next stage, the smart sensor/IoT includes additional computing resources to process and interpret data and make decisions locally. Thus “smart” functions are added successively to the conventional sensor [1,4].

### 1.2. Basic Resources of a Typical Smart Sensor/IoT Device and Their Limitations

Despite the nuances and differences in the definitions and characteristics of various devices, their main distinguishing feature is their limited resources.

A typical autonomously powered smart sensor/IoT device includes two basic groups of resources. *Hardware resources*: data storage, processing, and communication resources, as well as power supply. *Software resources*: operating systems, system software, preloaded applications, and lightweight services. The optimal distribution of these resources is crucial in most applications [5,6]. 

#### 1.2.1. Limitations Based on Hardware

(1) Energy and computational constraints:

Usually, IoT devices are battery-powered and typically use low-power processors. Therefore, computationally extensive algorithms that require significant resources cannot be transferred directly to such devices.

(2) Memory constraints: IoT devices are made with limited RAM and Flash memory compared to conventional digital systems (e.g., desktop computers, laptops, etc.). They usually use mobile lightweight software tools and operating systems. Therefore, calculation schemes require approaches for efficient memory use. However, the traditional computational algorithms are not specifically designed for this purpose and that is why such algorithms cannot be used directly for IoT devices.

#### 1.2.2. Limitations Based on Software

(1) Firmware constraints: The sensor–IoT operating systems that are embedded in the devices have thin stacks of network protocols.
(2) Flexibility: Remote reprogramming may not always be possible for devices, as the operating system may not be able to receive and integrate a new code.

### 1.3. Sensor Characteristics Linearization

#### 1.3.1. Sensor Transfer Functions 

A sensor device generates an output signal in response to a specific measurand/stimulus (input) by activating one or more physical effects (ergo, Seebeck’s effect, piezoresistive effect, etc.). The output from a sensor is usually an electrical quantity (e.g., voltage, current, frequency), denoted here by y, while the input, x, is some physical quantity of interest (e.g., temperature, chemical concentration, pressure). If the input–output function is time-invariant, it is commonly called a static transfer function/characteristic or simply a transfer function.

The sensors are parts of a measuring system. The main function of these systems is to derive the unknown value x from the measured value y. Thus, the measurement system shall employ an inverse sensor transfer function x=x(y) to obtain (calculate) the value of the stimulus x [7].

**Figure 1:**
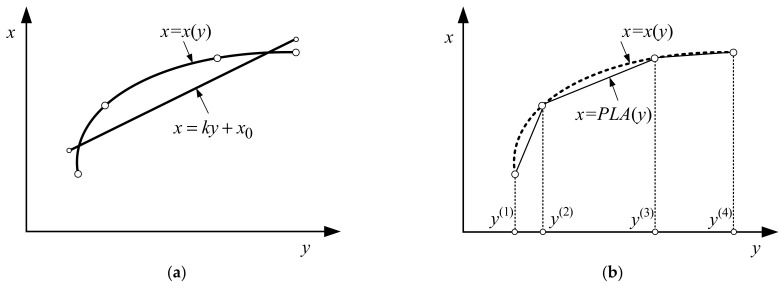
Graphical representation of inverse sensor transfer functions: (**a**) inverse sensor transfer function x=x(y) and its linear approximation $x=ky+x_{0}$; (**b**) inverse sensor transfer function x=x(y) and its piecewise linear approximation x=PLA(y).

In the context of the system (control) theory, the dependence y=y(x) represents the nonlinear scalar-valued characteristic of the sensor while its inverse x=x(y) represents the *inverse transfer function of the sensor* (Figure 1a)

#### 1.3.2. Approaches for Sensor Characteristics Linearization

Linearization is a major step in sensor signal processing. It can be considered as a nonlinearity correction between the output signal of the sensor and the associated measured quantity. The nonlinearity of the sensor can be reduced by using linearization schemes or a linearization algorithm [8]. Linearization methods can be grouped into three main classes:

- analog hardware linearization circuits;
- software-based algorithms for linearization;
- analog–digital mixed approaches [9].

An analog hardware method of linearization is performed by connecting a nonlinear analog circuit between the sensor and the ADC [10].

Software-based methods require the use of PCs or DSPs with significant computing resources [11]. Using these methods on low-cost controllers that support only simple mathematical operations is a major challenge due to the limited resources of the controller. Various software-based linearization methods are discussed in the literature. The most applied method is linearization based on the traditional look-up table (LUT), which can be implemented on any microcontroller [12]. In this method, the result of digital processing is used as an index in an array that stores the corrected data points [9,13].

The linearized sensor characteristics can greatly simplify the design, calibration, and measurement accuracy. This work elaborates further on the method presented in [14], used for the adaptive linear approximation of sensor characteristics.

In general, a scalar-valued inverse sensor transfer function is mathematically represented by a nonlinear regression model (e.g., exponential, power-law, or polynomial) constructed through least-squares error minimization (fit) of a statistically representative experimental data set. The identification of the inverse transfer function is often complicated and unreliable due to uncertainty in the choice of the analytic form of the function and reductions in its parametrization. This of course leads to unwanted errors in the calibrated sensor response and hence should be avoided [15]. 

A way to avoid the uncertainty issues emerging from nonlinear regression-based identification of the inverse sensor response is provided by piecewise linearization (segmentation) of its transfer function. The latter is essentially a (simplified) polygonal approximation of x=x(y) with algorithmic control of the approximation error (Figure 1b). 

#### 1.3.3. Piecewise Linear Approximation of Sensor Transfer Functions

Analog methods for linear approximation are still popular in engineering practice. However, thanks to advances in digital technology, the implementation of software methods can be completed easily, fast, at a low cost, and with a guarantee of high accuracy. Therefore, digital methods combined with software technologies solve linearization problems with better flexibility and efficiency.

Over the last two decades, different piecewise linear approximation (PLA) techniques have been proposed for the representation of sensor transfer functions. These techniques are reported in various application areas of sensor technology, such as data mining [16], data collection in wireless sensor networks [17,18], and activity recognition applications with wearable sensor nodes [19,20,21]. 

PLA algorithms for data volume reduction by approximating the signals collected in wireless sensor networks (WSN) are presented in [16,17]. These algorithms use buffers, and the calculation time depends on the length of the buffer. In [22], a fast alternative for time series segmentation is considered, in which the reduction in the calculation time significantly increases at the expense of the used memory. This is not an appropriate approach for resource-constrained architectures such as mobile sensor nodes. In contrast, the proposed approach has constant computation and memory complexity.

The SWAB (Sliding Window and Bottom-Up) algorithm is proposed in [16]. The approach is a combination of two steps, the first of which is offline segmentation. Based on this segmentation, in the second step, an online algorithm is created. An optimization of this algorithm, called mSWAB, is presented in [19]. A further improved implementation (called emSWAB) for limited resources, especially suitable for mobile sensor systems is presented in [20]. These approaches are based on linear interpolation, while the approach proposed in the paper is based on linear regression and can also be performed in offline and online modes.

In [23] another segmentation approach was introduced, based on polynomial least-squares approximation with polynomials of arbitrary order. From the presented experiments with this approach when first-order polynomials are used, results like the Sliding Window (SW) method are obtained in terms of the approximation error and the degree of data compression.

Another buffer-based PLA algorithm is presented in [24]. The proposed approach is a combination of either connected or disconnected segments. In contrast, the proposed algorithm for approximating the sensor characteristics uses connected segments, which is a more appropriate solution to be implemented in architectures with constraint memory and processing resources.

In addition to providing a reliable representation of transfer function, PLA approaches have two more attractive features:

a. They create a simple and fast-to-identify characteristic shape of the transfer function (see [19,21,25]) that could be used for just-in-time classification purposes in large-scale control systems.
b. They require a reduced amount of memory for data storage of the signal from the sensor device and hence possess significant economic potential for realization in smart sensors with reliable, low-cost microcontrollers. This is a crucial issue for two application domains: data mining when huge amounts of sensor data should be preprocessed under the constraint of short system-response times; and acquisition followed by wireless transmission of long-term sensor data.

### 1.4. Main Error Components of the Smart Sensor/IoT Device

**Figure 2:**
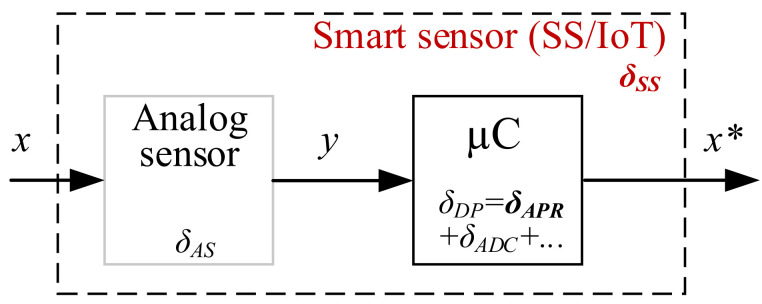
The basic structure and main error components of the smart sensor/IoT (after piecewise linear approximation and analog–digital conversion; x*—microcontroller output value).

As already mentioned, the goal of this study is to limit the memory footprint without violating system accuracy requirements. The basic structure of the smart sensor and its main components are shown in Figure 2. To achieve the required accuracy levels for a smart sensor, the main components of the compound must be considered. They can be divided into error components from the analog part $\delta_{AS}$ and components from the digital part $\delta_{DP}$. Details are given in [26].

Here $\delta_{AS}$—relative compound analog sensor error; $\delta_{DP}$—relative compound digital processing error caused by the digital domain signal; $\delta_{APR}=\frac{\Delta^{x}}{x_{FS}}$—relative approximation error; $\Delta^{x}$—predefined absolute *approximation* error; $x_{FS}$—full scale of microcontroller output value x*; $\delta_{ADC}$—relative analog–digital conversion error. Based on the considered serial structure, the total system error budget can be determined as:

$$\delta_{SS}=\sqrt{\delta_{AS}^{2}+\delta_{DP}^{2}}.$$

The main components of the digital processing error $\delta_{DP}$ can also be attributed to $\delta_{ADC}$ and $\delta_{APR}$. When an analog sensor and ADC are selected, the error levels $\delta_{AS}$ and $\delta_{ADC}$ cannot be affected. High-resolution ADCs are most often used in smart sensors and IoT implementation and therefore the $\delta_{ADC}$ the component is negligibly small which proves $\delta_{APR}$ to be a key component through which the total error levels can be managed, which will be discussed below.

To achieve typical goals in system design, where $\delta_{SS}\approx \delta_{AS}$, the aim is to have: $\delta_{AS}>\left(3÷10\right)\delta_{DP}$.

Published look-up tables, which are used in several applications, are often limited in the number of digits and this can be an additional source of rounding errors [26].

### 1.5. Proposed Approach

The proposed approach can be used for piecewise adaptive linearization of piecewise convex/concave sensor characteristics. As the name suggests, a typical piecewise convex/concave sensor response is characterized by a function consisting of a finite number of linked convex/concave segments with a strictly monotonous slope and hence a constant sign of the second derivative. Within the full measurement range of the sensor, the bounds of these segments can be identified from the condition of removing the second derivative, i.e., as the inflection points of the sensor characteristics.

**Figure 3:**
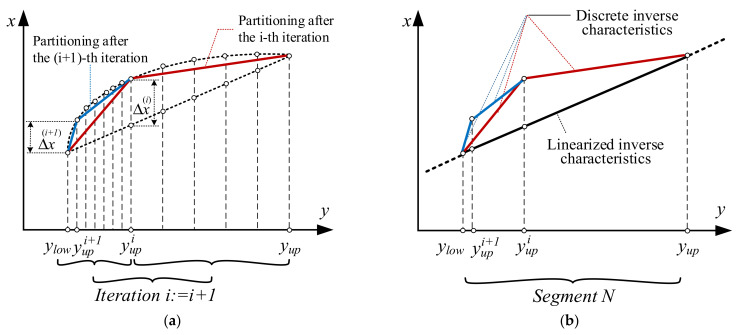
Graphical illustration of (**a**) the method in [11] and (**b**) the proposed approach.

The specificity of the proposed approach in comparison with the approach presented in [11] is illustrated in Figure 3.

An example of such a sensor characteristic is the relationship between resistance and temperature in the platinum temperature sensors set by the Callendar–van Dusen equation.

In [11,27,28], a linearization approach to sensor characteristics was developed in cases where the characteristic is a differentiable function. Its inverse feature is determined in a discrete form (freeform) based on an iterative algorithm with defined sensor characteristics and maximum approximation error. After that, based on the developed adaptive approach with a defined maximum approximation error, the discrete inverse sensor characteristic is replaced by a new discrete characteristic (also freeform), aimed at minimizing the number of points defining the characteristic, which in turn is related to the possibilities of using cheaper microcontrollers.

With the specified sensor, the transfer characteristic is of the type y=y(x) and the first task is to determine the inverse transfer sensor characteristic x=x(y). In cases where the inverse function cannot be obtained in an explicit form, due to the nonlinear relation between y(x) and x, using a discrete linear change of x, the corresponding values of y(x) are obtained, which upon receipt of the inverse function results in a discrete relationship between y(x) and x, but with a nonlinear discrete change of argument s. This problem can be solved by the splitting method [3], the Newton method [29], as well as by the iterative algorithm proposed in [11,30]. As a result, given a maximum approximation error, the inverse sensor characteristic is obtained in a discrete form $x_{i}=x_{i}\left(y_{i}\right),i=\bar{1,n}$, with a uniform changing step of the argument s.

The second task is related to the approximation of the obtained discrete inverse sensor characteristic in the form of $x_{i}=x_{i}\left(y_{i}\right),i=\bar{1,n}$, a new characteristic $x_{j}=x_{j}\left(y_{j}\right),j=\bar{1,k}$ with a specified maximum approximation error and minimization of k. One approach for solving the problem was developed in [2,11].

The purpose of the present work is to develop a new approach for solving the task of linearization of sensor characteristics (differentiable functions with a non-changing sign of curvature), which simultaneously solves the tasks of finding the inverse sensor characteristic and its linearization.

## 2. The Analytical Framework of the Proposed Approach for Linearization of Sensor Characteristics

Let a differentiable sensor characteristic of the type y=y(x), $x\in \left[x_{A_{i}},x_{B}\right]$, $i=\bar{1,n-1}$, be given. For each such sensor, the characteristic is fulfilled $y^{\prime }\left(x\right)\neq 0$, as the function y=y(x) is strictly monotonous ($y^{\prime }\left(x\right)>0$ or $y^{\prime }\left(x\right)<0$).

In the case considered here, if the sign of curvature remains unchanged, one of the following two conditions should be satisfied: $sign(y^{\prime \prime }\left(x\right))>0$ or $sign(y^{\prime \prime }\left(x\right))<0$.

**Figure 4:**
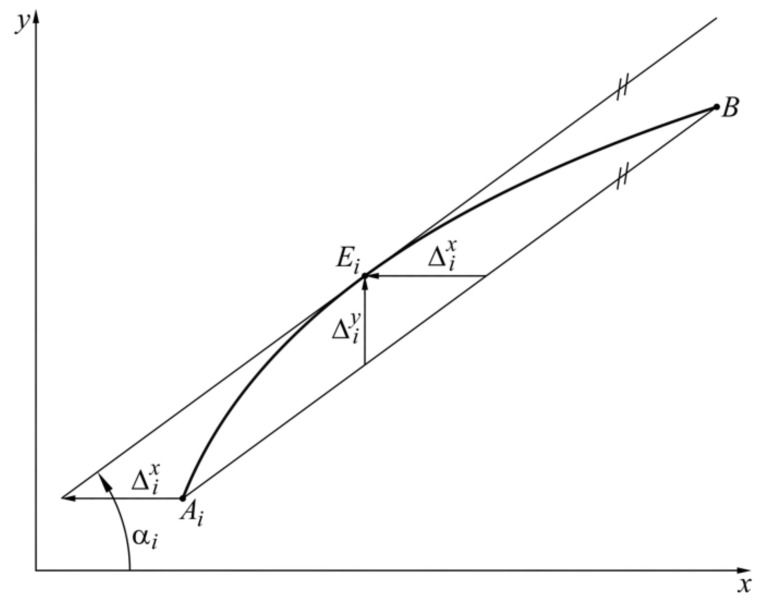
Graphical representation of the proposed approach.

Under linear approximation of the function within the specified range, the error $\Delta_{i}^{y}\left(x\right)$ is determined by

(1) $$\Delta_{i}^{y}\left(x\right)=y\left(x\right)-\left(y_{A_{i}}+\frac{y_{B}-y_{A_{i}}}{x_{B}-x_{A_{i}}}\left(x-x_{A_{i}}\right)\right)=y\left(x\right)-y_{A_{i}}-k_{i}\left(x-x_{A_{i}}\right),$$

where $k_{i}=\frac{y_{B}-y_{A_{i}}}{x_{B}-x_{A_{i}}}$ is the slope of the straight line $A_{i}B$ (Figure 4).

From the necessary condition for extremum, ${\left[\Delta_{i}^{y}\left(x_{E_{i}}\right)\right]}^{\prime }=0$ the following is obtained:

(2) $$y^{\prime }\left(x_{E_{i}}\right)-k_{i}=0,$$

as the condition is also sufficient, $y^{\prime \prime }\left(x\right)\neq 0$.

The extremum always exists (Role Theorem [31]—$\Delta_{i}^{y}\left(x_{A_{i}}\right)=\Delta_{i}^{y}\left(x_{B}\right)=0$), as at the same time it is unique—the function $\left(y^{\prime }\left(x\right)-k_{i}\right)$ is monotonous and has different signs at the ends of the range.

With Equation (1), when $x=x_{E_{i}}$, the extremum value is obtained

$$\Delta_{i}^{y}\left(x_{E_{i}}\right)=y\left(x_{E_{i}}\right)-y_{A_{i}}-k_{i}\left(x_{E_{i}}-x_{A_{i}}\right).$$

The extremum of the error $\Delta_{i}^{x}\left(y\right)$ at a linear approximation of the inverse function x(y) in the given range is determined by

$$\frac{\Delta_{i}^{y}\left(x_{E_{i}}\right)}{\Delta_{i}^{x}\left(x_{E_{i}}\right)}=-\tan\alpha_{i}=-k_{i},$$

under which

(3) $$\Delta_{i}^{x}\left(x_{E_{i}}\right)=\frac{\Delta_{i}^{y}\left(x_{E_{i}}\right)}{-k_{i}}=x_{E_{i}}-x_{A_{i}}-\frac{y\left(x_{E_{i}}\right)-y_{A_{i}}}{k_{i}}.$$

In the context of this work, of practical interest is the following problem case—the maximum approximation error $\Delta^{x}$ is specified, the absolute value of which is less than the absolute value of the defined by Equation (3) error in the given range $\left[x_{A_{i}},x_{B}\right]$. The goal is to find a subrange $\left[x_{A_{i}},x_{A_{i+1}}\right]$, where $\Delta_{i}^{x}\left(x_{E_{i}}\right)=\Delta^{x}$ is fulfilled.

From Equations (2) and (3) it follows that

$$\Delta_{i}^{x}\left(x_{E_{i}}\right)=\Delta^{x}=x_{E_{i}}-x_{A_{i}}-\frac{y\left(x_{E_{i}}\right)-y_{A_{i}}}{k_{i}}=x_{E_{i}}-x_{A_{i}}-\frac{y\left(x_{E_{i}}\right)-y_{A_{i}}}{y^{\prime }\left(x_{E_{i}}\right)},$$

respectively,

(4) $$y^{\prime }\left(x_{E_{i}}\right)-\frac{y\left(x_{E_{i}}\right)-y_{A_{i}}}{x_{E_{i}}-x_{A_{i}}-\Delta^{x}}=0.$$

The last equation defines $x_{E_{i}}$, then the right end $x_{A_{i+1}}$ of the range is determined by the equation

(5) $$y\left(x_{A_{i+1}}\right)=y_{A_{i}}+y^{\prime }\left(x_{E_{i}}\right)\left(x_{A_{i+1}}-x_{A_{i}}\right).$$

## 3. Essence of the Proposed Approach for Linearization of Sensor Characteristics

Let a differentiable sensor characteristic in the form y=y(x), $x\in \left[x_{A_{i}},x_{B}\right]$, $i=\bar{1,n-1}$ be given as the sign of curvature does not change $\left(sign(y^{\prime \prime }\left(x\right)\right)>0$ or $sign(y^{\prime \prime }\left(x\right))<0$). The aim is to obtain the inverse sensor characteristic x(y) in the linearized form at a specified maximum approximation error $\Delta^{x}$.

The approach is realized in the following way. With Equation (3) the extremum value of the $\Delta_{1}^{x}$ in the range $x\in \left[x_{A_{1}},x_{B}\right]$ is determined. If $\left|\Delta_{1}^{x}\right|<\left|\Delta^{x}\right|$ then the function is approximated throughout the range with segment $A_{1}B$ and the procedure ends. In the opposite case with Equations (4) and (5), the subrange $x\in \left[x_{A_{1}},x_{A_{2}}\right]$, where $\Delta_{1}^{x}=\Delta^{x}$ is determined.

Then, through Equation (3), the extremum value of $\Delta_{2}^{x}$ in the range $x\in \left[x_{A_{2}},x_{B}\right]$ is determined. If $\left|\Delta_{2}^{x}\right|<\left|\Delta^{x}\right|$, then the function is approximated throughout the range with the segment $A_{2}B$ and the procedure ends, or otherwise through Equations (4) and (5) the subrange $x\in \left[x_{A_{2}},x_{A_{3}}\right]$, where $\Delta_{2}^{x}=\Delta^{x}$, is determined, etc.

The inverse sensor characteristic x(y) is approximated by the broken line $A_{1}A_{2}A_{3}\ldots A_{n-1}B\equiv A_{n}$, $A_{i}\left(y_{A_{i}},x_{A_{i}}\right)$, $i=\bar{1,n-1}$, $A_{n}\left(y_{B},x_{B}\right)$, as

(6) $$x\left(y\right)=x_{A_{i}}+\left(y-y_{A_{i}}\right)\frac{x_{A_{i+1}}-x_{A_{i}}}{y_{A_{i+1}}-y_{A_{i}}},\;y\in \left[y_{A_{i}},y_{A_{i+1}}\right],\;i=\bar{1,n-1}.$$

Generally, in all intervals, except for the last one, the maximum approximation errors are equal and equal to the maximum specified approximation error $\Delta^{x}$. The error $\Delta_{i}^{x}\left(x\right)$ in each of the intervals is determined by the following equation:

(7) $$\Delta_{i}^{x}\left(x\right)=x-x_{A_{i}}-\left(y\left(x\right)-y_{A_{i}}\right)\frac{x_{A_{i+1}}-x_{A_{i}}}{y_{A_{i+1}}-y_{A_{i}}},\;\;x\in \left[x_{A_{i}},x_{A_{i+1}}\right],\;i=\bar{1,n-1}.$$

Considering the next equation

(8) $$\Delta_{i}^{x}\left[y\left(x\right)\right]=\Delta_{i}^{x}\left(x\right),\;x\in \left[x_{A_{i}},x_{A_{i+1}}\right],\;i=\bar{1,n-1},$$

and Equation (7) the error $\Delta_{i}^{x}\left(y\right)$ can be found. 

**Illustrative example:** At a specified maximum approximation error $\Delta^{x}$, find the linearized inverse characteristic of the characteristic $y\left(x\right)=a_{1}+a_{2}{\left(x-a_{3}\right)}^{2}$, $x\in \left[x_{A},x_{B}\right]$, when $\Delta^{x}=2,$ $a_{1}=2,$ $a_{2}=0.02$, $a_{3}=2$, $x_{A}=3$, $x_{B}=51$.

Then $y\left(x\right)=2+0.02{\left(3-2\right)}^{2}=2.02$, and $y^{\prime }\left(x\right)=0.04\left(x-2\right)$, $y^{\prime \prime }\left(x\right)=0.04$. So $y\left(x_{A_{i}}\right)$ = 2 + 0.02. 

From (4) follows

$$0.04\left(x_{E_{i}}-2\right)-\frac{2+0.02{\left(x_{E_{i}}-2\right)}^{2}-2.02}{x_{E_{i}}-3-2}=0,\;$$

respectively, $x_{E_{i}}^{2}-10x_{E_{i}}+17=0$

The first of the two solutions 2.171572875 does not belong to the considered interval ($2.171572875<x_{A_{i}}=3$). Only the second solution 7.828427125 belongs to the considered interval. Therefore, $x_{E_{i}}=7.828427125$.

From (5) follows

$$2+0.02{\left(x_{A_{i+1}}-2\right)}^{2}=2.02+0.04\left(7.828427125-2\right)\left(x_{A_{i+1}}-3\right),\;$$

Respectively,

$$x_{A_{i+1}}^{2}-15.65685425x_{A_{i+1}}+37.97056275=0.$$

The first of the two solutions (3 and 12.65685425) corresponds to the starting point. Therefore, $x_{A_{i}}=3$ and $x_{A_{i}}=12.65685425$.

**Figure 5:**
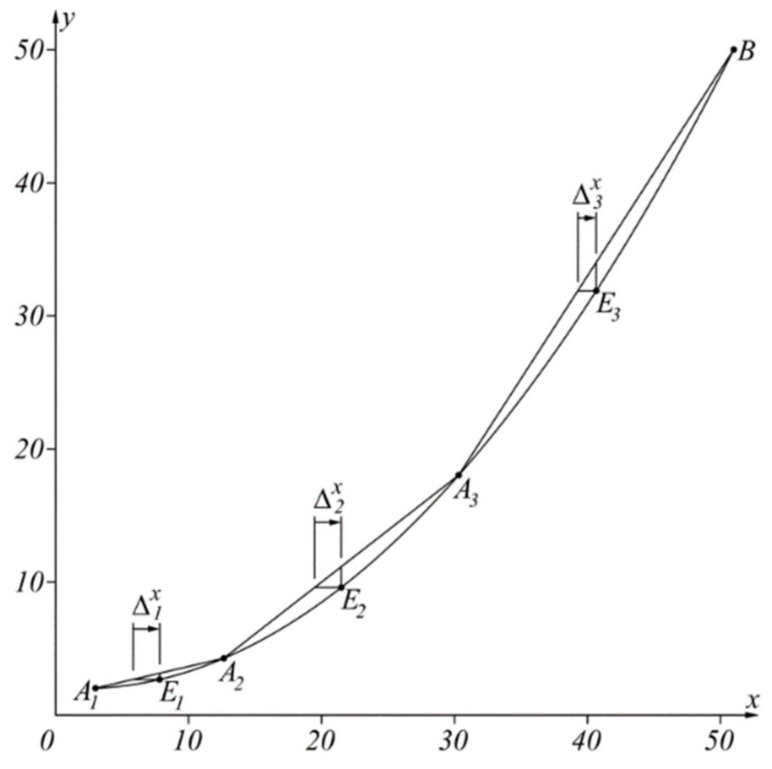
Graphical results of the solution of the illustrative example.

As a result of this approach, the inverse sensor characteristic x(y) is approximated by the broken line $A_{1}A_{2}A_{3}B$, as shown in Table 1, where the coordinates of the points are given, also the corresponding intervals of the argument variation, the values of the argument in the extrema in the corresponding interval (as a result of the properties of the square parabola they are always in the middle of the interval) and the corresponding error $\Delta_{i}^{x}$. The illustrative example is selected to enable good graphical visualization of the results shown in Figure 5.

## 4. Linearization of the Inverse Sensor Characteristics of Temperature Sensors 

The relation between the resistance and the temperature in the platinum temperature sensors is set by the equation of Callendar–van Dusen [32,33]. Using the proposed approach, the task of interval linearization of the inverse characteristic will be solved.

### 4.1. Linearization of the Inverse Sensor Characteristics of the Platinum Temperature Sensors in the Range T∈[0 , 661 ] °C

The Callendar–van Dusen equation in the case under consideration is given by

(9) $$R_{T}=R_{0}\left(1+AT+BT^{2}\right),$$

where

*T*, °C—temperature;
$R_{T}$—measured resistance at temperature T;
$R_{0}$—measured resistance at temperature T=0 °C;
$A=3.908310^{-3}$; $B=-5.77510^{-7}$—coefficients according to ITS 90/IEC 60751 [34,35].

Under the denotations $R\left(T\right)=\frac{R_{T}}{R_{0}}$ and $a_{3}=\frac{-A}{\left(2B\right)}$, Equation (9) takes the form

(10) $$R\left(T\right)=1-\frac{A^{2}}{4B}+B{\left[T+\frac{A}{2B}\right]}^{2}=1-\frac{A^{2}}{4B}+B{\left(T-a_{3}\right)}^{2}.$$

Differentiating (10) twice, concerning temperature T, leads to the first and second derivatives, respectively,

(11) $$R^{\prime }\left(T\right)=2B\left(T-a_{3}\right),$$

(12) $$R^{\prime \prime }\left(T\right)=2B.$$

**Figure 6:**
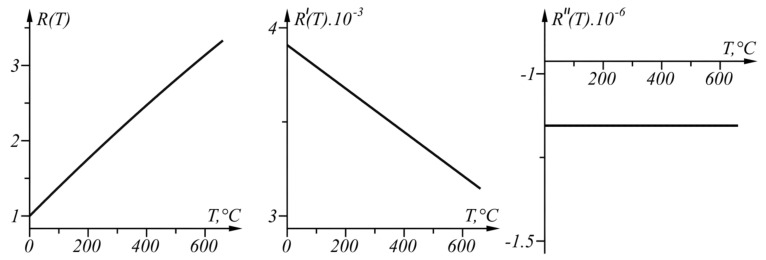
Graphs of the functions R(T), $R^{\prime }\left(T\right)$, and $R^{\prime \prime }\left(T\right)$ in the range T∈[0, 661] °C.

The graphs of function R(T), its first derivative $R^{\prime }\left(T\right)$, and its second derivative $R^{\prime \prime }\left(T\right)$ in the range T∈[0, 661] °C defined by Equations (10)–(12), respectively, are shown in Figure 6.

Since $R^{\prime \prime }\left(T\right)$ does not change its sign (the curve is concave in the considered range), the proposed approach for linear interval linearization can be applied because the function T(R) can be found in the linearized form.

Equations (2)–(5) and (7), required for the approach, accordingly take the form:

(13) $$R^{\prime }\left(T_{E_{i}}\right)-k_{i}=2B\left(T_{E_{i}}-a_{3}\right)-\frac{R_{B}-R_{A_{i}}}{T_{B}-T_{A_{i}}}=0\;\mathrm{or}\;T_{E_{i}}=\frac{T_{A_{i}}+T_{B}}{2};\;$$

(14) $$\Delta_{i}^{T}\left(T_{E_{i}}\right)=\frac{\Delta_{i}^{R}\left(T_{E_{i}}\right)}{-k_{i}}=T_{E_{i}}-T_{A_{i}}-\frac{R\left(T_{E_{i}}\right)-R_{A_{i}}}{k_{i}}=T_{E_{i}}-T_{A_{i}}-\frac{\left(R\left(T_{E_{i}}\right)-R_{A_{i}}\right)\left(T_{B}-T_{A_{i}}\right)}{R_{B}-R_{A_{i}}}\;=\left(T_{B}-T_{A_{i}}\right)\left(\frac{1}{2}-\frac{1}{4}\frac{T_{B}+3T_{A_{i}}-4a_{3}}{T_{B}+T_{A_{i}}-2a_{3}}\right);$$

(15) $$T_{E_{i}}=\Delta^{T}+T_{A_{i}}+\sqrt{{\left(\Delta^{T}+T_{A_{i}}\right)}^{2}-T_{A_{i}}{}^{2}-2a_{3}\Delta^{T}};$$

(16) $$T_{A_{i+1}}=2T_{E_{i}}-T_{A_{i}}.$$

(17) $$\Delta_{i}^{T}\left(T\right)=T-T_{A_{i}}-\left(R\left(T\right)-R_{A_{i}}\right)\frac{T_{A_{i+1}}-T_{A_{i}}}{R_{A_{i+1}}-R_{A_{i}}},\;x\in \left[T_{A_{i}},T_{A_{i+1}}\right],\;i=\bar{1,n-1}.$$

**Figure 7:**
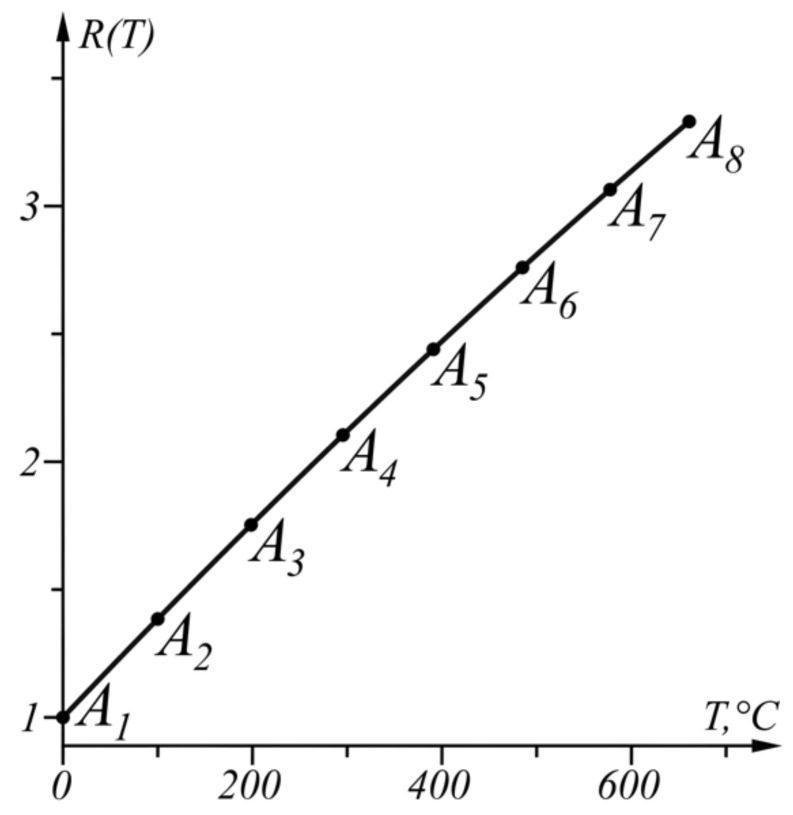
Graphical results of the solution in the range T∈[0, 661] °C.

**Figure 8:**
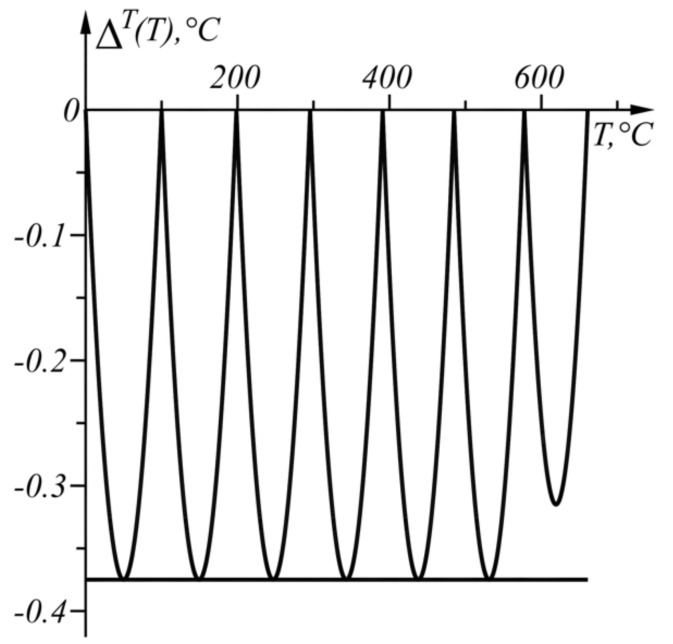
Graph of the function $\Delta^{T}\left(T\right)$ in the range T∈[0, 661] °C.

The numerical results from the application of the approach, when $\Delta^{T}=-0.375\;{}^{\circ}C$ in the given temperature range $T_{A_{1}}=0\;{}^{\circ}C$, $T_{B}=661\;{}^{\circ}C$, are shown in Table 2 and in graphical form in Figure 7. The graph of the function $\Delta^{T}\left(T\right)$ from (17) is shown in Figure 8.

### 4.2. Linearization of the Inverse Sensor Characteristics of the Platinum Temperature Sensors in the Range T∈[−200,0] °C

The Callendar–van Dusen equation when considering the case is given by

(18) $$R_{T}=R_{0}\left(1+AT+BT^{2}-100CT^{3}+CT^{4}\right),$$

where

T, °C—temperature;
$R_{T}$—measured resistance at temperature *T*;
$R_{0}$—measured resistance at temperature *T* = 0 °C;
$A=3.908310^{-3}$; $B=-5.77510^{-7}$; $C=-4.18310^{-12}$—coefficients according to ITS 90/IEC 60751 [34].

Under the denotation, $R\left(T\right)=\frac{R_{T}}{R_{0}}$, the Equation (18) takes the form

(19) $$R\left(T\right)=1+AT+BT^{2}-100CT^{3}+CT^{4}.$$

Differentiating (19) twice, concerning the temperature T, leads to the first and second derivatives, respectively,

(20) $$R^{\prime }\left(T\right)=A+2BT-300CT^{2}+4CT^{3};$$

(21) $$R^{\prime \prime }\left(T\right)=2B-600CT+12CT^{2}.$$

**Figure 9:**
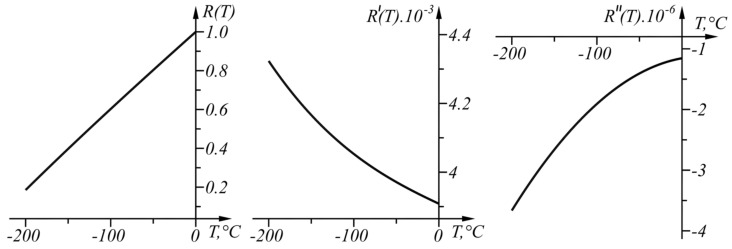
Graphs of the functions *R*(*T*), $R^{\prime }\left(T\right)$, and $R^{\prime \prime }\left(T\right)$ in the range T∈[−200,0] °C.

The graphs of R(T), $R^{\prime }\left(T\right)$, and $R^{\prime \prime }\left(T\right)$ are shown in Figure 9.

Since $R^{\prime \prime }\left(T\right)$ does not change its sign (the curve is concave in the range considered), the proposed approach for linear interval linearization can be applied because function T(R) can be found in the linearized form.

Equations (2)–(5) needed for the implementation of the approach take the form, respectively,

(22) $$A+2BT_{E_{i}}-300CT_{E_{i}}{}^{2}+4CT_{E_{i}}{}^{3}-\frac{R_{B}-R_{A_{i}}}{T_{B}-T_{A_{i}}}=0,$$

(23) $$\Delta_{i}^{T}\left(T_{E_{i}}\right)=\frac{\Delta_{i}^{R}\left(T_{E_{i}}\right)}{-k_{i}}=T_{E_{i}}-T_{A_{i}}-\frac{R\left(T_{E_{i}}\right)-R_{A_{i}}}{k_{i}}=\;T_{E_{i}}-T_{A_{i}}-\left(A\left(T_{E_{i}}-T_{A_{i}}\right)+B\left(T_{E_{i}}{}^{2}-T_{A_{i}}{}^{2}\right)-100C\left(T_{E_{i}}{}^{3}-T_{A_{i}}{}^{3}\right)+C\left(T_{E_{i}}{}^{4}-T_{A_{i}}{}^{4}\right)\right)\frac{T_{B}-T_{A_{i}}}{R_{B}-R_{A_{i}}}.$$

(24) $$R^{\prime }\left(T_{E_{i}}\right)-\frac{R\left(T_{E_{i}}\right)-R_{A_{i}}}{T_{E_{i}}-T_{A_{i}}-\Delta^{T}}=A+2BT_{E_{i}}-300CT_{E_{i}}{}^{2}+4CT_{E_{i}}{}^{3}\;-\frac{A\left(T_{E_{i}}-T_{A_{i}}\right)+B\left(T_{E_{i}}{}^{2}-T_{A_{i}}{}^{2}\right)-100C\left(T_{E_{i}}{}^{3}-T_{A_{i}}{}^{3}\right)+C\left(T_{E_{i}}{}^{4}-T_{A_{i}}{}^{4}\right)}{T_{E_{i}}-T_{A_{i}}-\Delta^{T}}=0.$$

(25) $$1+AT_{A_{i+1}}+BT_{A_{i+1}}{}^{2}-100CT_{A_{i+1}}{}^{3}+CT_{A_{i+1}}{}^{4}=1+AT_{A_{i}}+BT_{A_{i}}{}^{2}-100CT_{A_{i}}{}^{3}+\;+CT_{A_{i}}{}^{4}+\left(A+2BT_{E_{i}}-300CT_{E_{i}}{}^{2}+4CT_{E_{i}}{}^{3}\right)\left(T_{A_{i+1}}-T_{A_{i}}\right).$$

The proposed approach will be applied when $\Delta^{T}=-0.375\;{}^{\circ}\text{C}$ in the temperature range $T_{A_{1}}=0\;{}^{\circ}\text{C}$, $T_{B}=-200\;{}^{\circ}\text{C}$.

Using Equation (22), the value of the argument $T_{E_{1}}^{\prime }$ is determined, under which the function $\Delta_{1}^{T}\left(R_{E}\right)$ (23) has an extremum in the range $T_{B}=-200\;{}^{\circ}\text{C}<T<T_{A_{1}}=0\;{}^{\circ}\text{C}$. 

The third power Equation (22) has two complex and one real root $T_{E_{1}}=-110.583\;{}^{\circ}\text{C}$. When $T_{E_{1}}=-110.583\;{}^{\circ}\text{C}$, $\Delta_{1}^{T}=-2.472\;{}^{\circ}\text{C}$ is obtained through Equation (23). As $\left|\Delta_{1}^{T}\right|>\left|\Delta^{T}\right|$, Equations (24) and (25) determine the subrange $T\in \left[T_{A_{2}},T_{A_{1}}\right]$ where $\Delta_{1}^{T}=\Delta^{T}$. The fourth power Equation (24) has two real and two complex roots as the real root $T_{E_{1}}=-47.977\;{}^{\circ}\text{C}$ belongs to the range $\left[T_{B},T_{A_{1}}\right]$. The Equation (25) is also a fourth power equation and has two real and two complex roots as one of the real roots is $T_{A_{1}}$ and the other one is $T_{A_{2}}=-92.292\;{}^{\circ}\text{C}$.

Then the same steps are applied to the range $T_{B}=-200{\;}^{\circ }C<T<T_{A_{2}}=-92.292\;{}^{\circ}\text{C}$. The Equation (22) has two complex and one real root $T_{E_{2}}=-149.314\;{}^{\circ}C$. When $T_{E_{2}}=-149.314\;{}^{\circ}\text{C}$, $\Delta_{2}^{T}=-0.915\;{}^{\circ}C$ is obtained by Equation (23). As $\left|\Delta_{2}^{T}\right|>\left|\Delta^{T}\right|$, Equations (24) and (25) define the subrange $T\in \left[T_{A_{3}},T_{A_{2}}\right]$ where $\Delta_{2}^{T}=\Delta^{T}$. Equation (24) has two real and two complex roots as one of the real roots belongs to the range $\left[T_{B},T_{A_{2}}\right]$ is $T_{E_{2}}=-130.207\;{}^{\circ}\text{C}$. Equation (25) has two real and two complex roots as one of the real roots is $T_{A_{2}}$ and the other one is $T_{A_{3}}=-165.17\;{}^{\circ}C$. 

Then, the same consecution is applied to the range $T_{B}=-200\;{}^{\circ}\text{C}<T<T_{A_{3}}=-165.178\;{}^{\circ}\text{C}$. The real root of Equation (22) is $T_{E_{3}}=-182.909\;{}^{\circ}\text{C}.$ When $T_{E_{3}}=-182.909\;{}^{\circ}\text{C}$, Equation (23) $\Delta_{T_{3}}=-0.117\;{}^{\circ}\text{C}$ is obtained. As $\left|\Delta_{T}\right|<\left|\Delta^{T}\right|$, the procedure ends.

**Figure 10:**
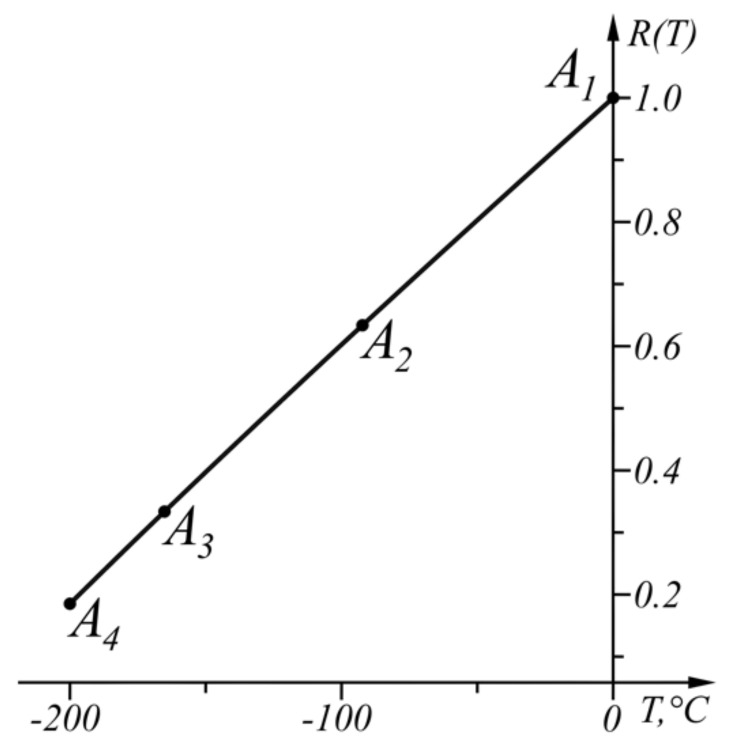
Graphical results of the solution in the range T∈[−200, 0] °C.

**Figure 11:**
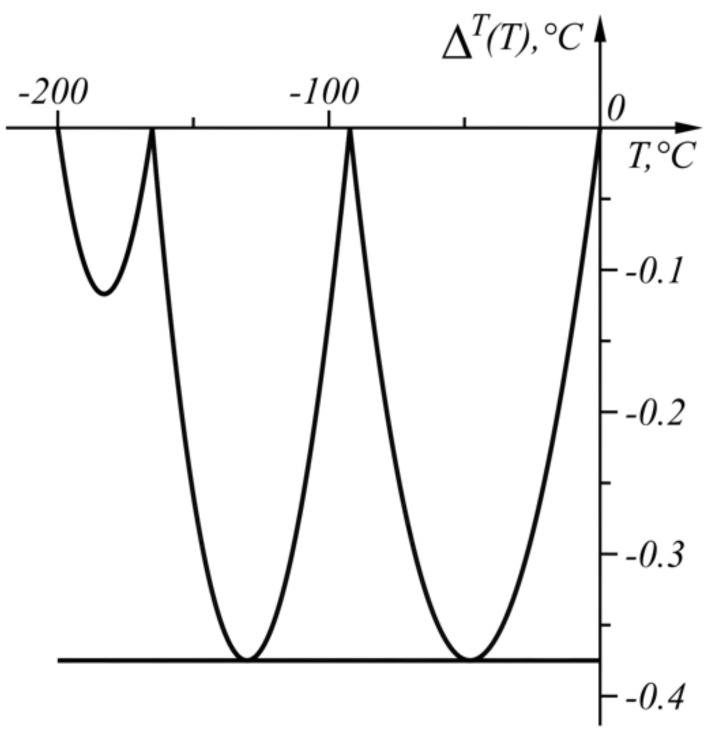
Graph of the function $\Delta^{T}\left(T\right)$ in the range T∈[−200, 0] °C.

The numerical results of the application of the approach when $\Delta^{T}=-0.375\;{}^{\circ}\text{C}$ are shown in Table 3 and in graphical form in Figure 10. The graph of the function $\Delta^{T}\left(T\right)$ (17) is shown in Figure 11.

### 4.3. Microcontroller Implementation of the Inverse Sensor Characteristics Linearization of Platinum Temperature Sensors in the Range T∈[0, 661] °C

This design uses a Lolin 32 development board based on Espressif Systems’ ESP32 microcontroller. Its main features include integrated Wi-Fi, flash memory, and USB communication, and is often used for IoT designs [36].

The linearization is performed by segmentation, in which the range is divided into the corresponding number of segments (from 1 to End) to guarantee the requirement for the maximum approximation error. The linearization algorithm for low-cost microcontrollers is presented in a pseudo-code form below (see Algorithm 1).
**Algorithm 1** Linearization**Input**: Measured RTD resistance, **R_T**, floating-point type **Output**: Calculated temperature, **Temperature**, floating-point
type*Initialization: defining the coordinates of the points bounding each interval Ra_1, Ra_2... Ra_End and Ta_1, Ta_2... Ta_End*
1: Determine the interval (n) in which
the measured resistance is located, and whether it is in the respective
temperature range
      If ((**R_T** > Ra_n) and (**R_T** ≤ Ra_(n + 1)))
2: Calculate the temperature according to the formula
      **Temperature** = (R_T − Ra_n) ∗ ((Ta_(n + 1) − Ta_n)/(Ra_(n + 1) − Ra_n)) +
Ta_n;
3: Return **Temperature**


The algorithm is implemented as a function in the Lolin D32 development system for the ESP32 microcontroller. When variables and constants are represented with a floating-point data type, the memory footprint of the function is 36 bytes of flash and 8 bytes of the system’s RAM. The function is compiled with both Os and g3 optimizations—for size and debug information, respectively.

For comparison, in an implementation based on an optimized lookup table using an unsigned integer, the occupied program memory is about 3k bytes and 8 bytes of RAM [37].

### 4.4. An Illustrative Example of Linearization of the Inverse Sensor Characteristics of K-Type Thermocouples in the Temperature Range T∈[−200, 0] °C and Maximum Approximation Error $\Delta^{T}=0.04\;{}^{\circ}\text{C}$

The thermoelectric voltage *E* in microvolts, as a function of temperature *T* in degrees Celsius, is defined by:

(26) $$E\left(t\right)=\overset{10}{\underset{1}{\sum }}c_{i}\;T^{i}\;,$$

where the coefficients $c_{i}$ are specified in NIST ITS-90 [38].

**Figure 12:**
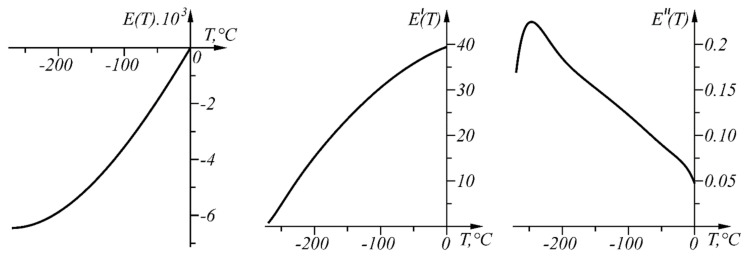
Graphs of the functions E(T), $E^{\prime }\left(T\right)$, and $E^{\prime \prime }\left(T\right)$ in the range T∈[−270, 0] °C.

The graphs of function E(T), its first derivative $E^{\prime }\left(T\right)$, and its second derivative $E^{\prime \prime }\left(T\right)$ in the range, T∈[−270, 0] °C, are shown in Figure 12.

Since $E^{\prime \prime }\left(T\right)$ does not change sign (the curve is convex in the considered range), the proposed approach for linear interval linearization can be applied and the sensor characteristic can be determined in linearized form.

**Figure 13:**
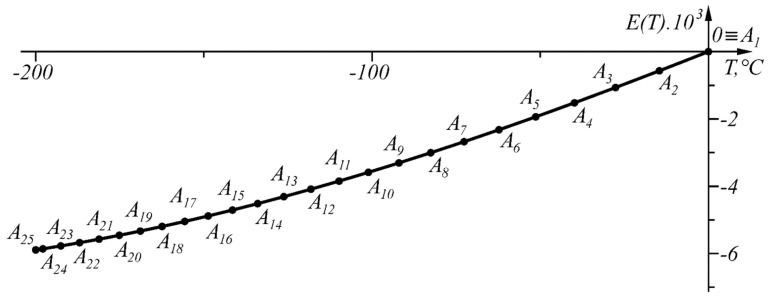
Graphical results of the solution in the range T∈[−200, 0] °C.

**Figure 14:**
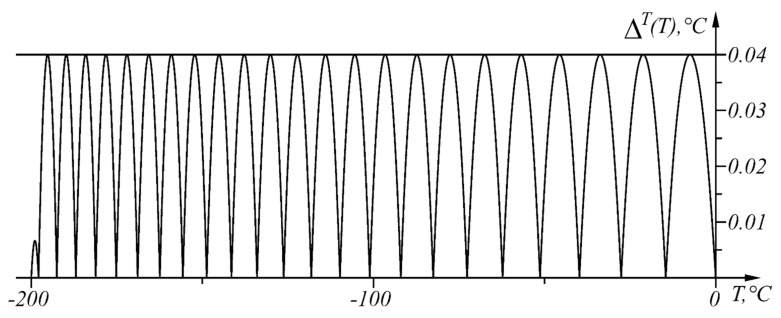
Graph of the function $\Delta^{T}\left(T\right)$ in the range T∈[−200, 0] °C.

The numerical results from the application of the approach, when $\Delta^{T}=0.04\;{}^{\circ}\text{C}$ in the given temperature range $T_{A_{1}}=0\;{}^{\circ}\text{C}$, $T_{B}=T_{A_{48}}=-200\;{}^{\circ}\text{C}$, are shown in Table 4 and in graphical form in Figure 13. The graph of the function $\Delta^{T}\left(T\right)$ is shown in Figure 14.

The linearization of the k-type thermocouple in the range [−200, 0] °C is implemented in the Lolin D32 development system for the ESP32 microcontroller. Variables and constants are represented by floating-point data type, the memory footprint of the function is about 145.3 kB, with activated size optimization “−Os”. This is about 10% of the system’s resources. 

## 5. Conclusions

This paper presented an approach for linear interval approximation of sensor characteristics y=y(x) where the characteristics are differentiable functions in which the sign of curvature does not change, i.e., they are either “concave” or “convex” functions. The aim is to obtain a discreet type of inverse sensor characteristic $x_{i}=x_{i}\left(y_{i}\right),i=\bar{1,n}$, with a predefined maximum approximation error $\Delta^{x}$, with minimization of the number of points defining the characteristic, which in turn is related to the possibilities for using cheaper microcontrollers. The approach is characterized by the fact that when the maximum approximation error $\Delta^{x}$ under the linearization of the inverse sensor characteristic is given, the inverse sensor characteristic $x_{i}=x_{i}\left(y_{i}\right),\;i=\bar{1,n}$ is found directly in the linearized form. The advantages of the developed approach are as follows:

- the approach is applied at intervals, with each subsequent step (each successive interval) resulting in a similar solution of the problem under the new initial conditions to obtain the directly sought solution, which in turn contains the initial conditions for the next step;
- the maximum error under linearization of the inverse sensor characteristic at all intervals, except in the general case of the last one, is the same;
- the approach allows that different maximum approximation errors are set at each subsequent interval;
- the approach allows the application to general types of differentiable sensor characteristics with piecewise concave/convex properties.

The proposed approach is verified through linearization of the inverse sensory characteristics of the Pt100 sensor and k-type thermocouple. The proposed approach to linear interval approximation of sensor characteristics y=y(x), which are differentiable functions, can be also extended to sensor characteristics with inflection points (characteristics where the sign of curvature changes, in some subintervals they are “concave”, and in others, they are “convex” functions). A typical example of such a sensor characteristic is the Type K Thermocouple. 

To overcome the problems arising from resource constraints, appropriate algorithms are needed that allow efficient connectivity and intelligent management of the measurement processes. Although the discussed computational examples are aimed at building adaptive approximations for temperature sensors, the algorithm can be applied easily to many other sensor types.

## Figures and Tables

**Table 1:** Results of the solution of the illustrative example.

$A_{1}A_{2}$	$A_{2}A_{3}$	$A_{3}B$
$A_{1}\left(3.000,\;2.020\right)$	$A_{2}\left(12.657,\;4.271\right)$	$A_{3}\left(30.314,\;18.033\right)$
$A_{2}\left(12.657,\;4.271\right)$	$A_{3}\left(30.314,\;18.033\right)$	$B\left(51.000,\;50.020\right)$
$x\in \left[3.000,\;12.657\right]$	$x\in \left[12.657,\;30.314\right]$	$x\in \left[30.314,\;51.000\right]$
$x_{E_{1}}=7.828$	$x_{E_{2}}=21.485$	$x_{E_{3}}=43.142$
$\Delta_{1}^{x}=2$	$\Delta_{2}^{x}=2$	$\Delta_{3}^{x}=1.384$

**Table 2:** Results in the range T∈[0, 661] °C.

$A_{1}A_{2}$	$A_{2}A_{3}$	$A_{3}A_{4}$	$A_{4}A_{5}$
$A_{1}\left(0,1\right)$	$A_{2}\left(100.007,\;1.385\right)$	$A_{3}\left(198.514,\;1.753\right)$	$A_{4}\left(295.521,\;2.105\right)$
$A_{2}\left(100.007,\;1.385\right)$	$A_{3}\left(198.514,\;1.753\right)$	$A_{4}\left(295.521,\;2.105\right)$	$A_{5}\left(391.028,\;2.440\right)$
$T\in \left[0,\;100.007\right]$	$T\in \left[100.007,\;198.514\right]$	$T\in \left[198.514,\;295.521\right]$	$T\in \left[295.521,\;391.028\right]$
$T_{E_{1}}=50.0035$	$T_{E_{2}}=149.261$	$T_{E_{3}}=247.018$	$T_{E_{4}}=343.275$
$\Delta_{1}^{T}=-0.375$	$\Delta_{2}^{T}=-0.375$	$\Delta_{3}^{T}=-0.375$	$\Delta_{4}^{T}=-0.375$
$A_{5}A_{6}$	$A_{6}A_{7}$	$A_{7}A_{8}$	
$A_{5}\left(391.028,\;2.440\right)$	$A_{6}\left(485.035,\;2.760\right)$	$A_{7}\left(577.543,\;3.065\right)$	
$A_{6}\left(485.035,\;2.760\right)$	$A_{7}\left(577.543,\;3.065\right)$	$A_{8}\equiv B\left(661,\;3.331\right)$	
$T\in \left[391.028,\;485.035\right]$	$T\in \left[485.035,\;577.543\right]$	$T\in \left[577.543,\;661\right]$	
$T_{E_{5}}=438.032$	$T_{E_{6}}=531.289$	$T_{E_{7}}=619.271$	
$\Delta_{5}^{T}=-0.375$	$\Delta_{6}^{T}=-0.375$	$\Delta_{7}^{T}=-0.315$	

**Table 3:** Results in the range T∈[−200, 0] °C.

$A_{1}A_{2}$	$A_{2}A_{3}$	$A_{3}A_{4}$
$A_{1}\left(0,1\right)$	$A_{2}\left(-92.292,0.634\right)$	$A_{3}\left(-165.178,\;0.334\right)$
$A_{2}\left(-92.292,0.634\right)$	$A_{3}\left(-165.178,\;0.334\right)$	$A_{4}\equiv B\left(-200,\;0.185\right)$
T∈[−92.292,0]	T∈[−165.178,−92.292]	T∈[−200,−165.178]
$T_{E_{1}}=-47.977$	$T_{E_{2}}=-130.207$	$T_{E_{3}}=-182.909$
$\Delta_{T_{1}}=-0.375$	$\Delta_{T_{2}}=-0.375$	$\Delta_{T_{3}}=-0.117$

**Table 4:** Results in the range T∈[−200, 0] °C.

$A_{1}A_{2}$	$A_{2}A_{3}$	$A_{3}A_{4}$	$A_{4}A_{5}$
$A_{1}\left(0,0\right)$	$A_{2}\left(-14.596,-569.9626\right)$	$A_{3}\left(-27.626,-1066.926\right)$	$A_{4}\left(-39.819,-1520.303\right)$
$A_{2}\left(-14.596,-569.9626\right)\;$	$A_{3}\left(-27.626,-1066.926\right)$	$A_{4}\left(-39.819,-1520.303\right)$	$A_{5}\left(-51.342,-1937.358\right)$
$T\in \left[-14.596,\;0\right]$	$T\in \left[-27.626,\;-14.596\right]$	$T\in \left[-39.819,\;-27.626\right]$	$T\in \left[-51.342,\;-39.819\right]$
$T_{E_{1}}=-7.494$	$T_{E_{2}}=-21.181929$	$T_{E_{3}}=-33.769$	$T_{E_{4}}=-45.620$
$\Delta^{T}=0.04$	$\Delta^{T}=0.04$	$\Delta^{T}=0.04$	$\Delta^{T}=0.04$
$A_{5}A_{6}$	$A_{6}A_{7}$	$A_{7}A_{8}$	$A_{8}A_{9}$
$A_{5}\left(-51.342,-1937.358\right)$	$A_{6}\left(-62.267,-2321.642\right)$	$A_{7}\left(-72.653,-2676.126\right)$	$A_{8}\left(-82.554,-3003.567\right)$
$A_{6}\left(-62.267,-2321.642\right)$	$A_{7}\left(-72.653,-2676.126\right)$	$A_{8}\left(-82.554,-3003.567\right)$	$A_{9}\left(-92.019,-3306.423\right)$
$T\in \left[-62.267,\;-51.342\right]$	$T\in \left[-72.653,\;-62.267\right]$	$T\in \left[-82.554,\;-72.653\right]$	$T\in \left[-92.019,\;-82.554\right]$
$T_{E_{5}}=-56.839$	$T_{E_{6}}=-67.489$	$T_{E_{7}}=-77.628$	$T_{E_{8}}=-87.308$
$\Delta^{T}=0.04$	$\Delta^{T}=0.04$	$\Delta^{T}=0.04$	$\Delta^{T}=0.04$
$A_{9}A_{10}$	$A_{10}A_{11}$	$A_{11}A_{12}$	$A_{12}A_{13}$
$A_{9}\left(-92.019,-3306.423\right)$	$A_{10}\left(-101.091,-3586.812\right)$	$A_{11}\left(-109.801,-3846.531\right)$	$A_{12}\left(-118.178,-4087.117\right)$
$A_{10}\left(-101.091,-3586.812\right)$	$A_{11}\left(-109.801,-3846.531\right)$	$A_{12}\left(-118.178,-4087.117\right)$	$A_{13}\left(-126.243,-4309.902\right)$
$T\in \left[-101.091,\;-92.019\right]$	$T\in \left[-109.801,-101.091\right]$	$T\in \left[-118.178,\;-109.801\right]$	$T\in \left[-126.243,\;-118.178\right]$
$T_{E_{9}}=-96.573$	$T_{E_{10}}=-105.461$	$T_{E_{11}}=-114.003$	$T_{E_{12}}=-122.223$
$\Delta^{T}=0.04$	$\Delta^{T}=0.04$	$\Delta^{T}=0.04$	$\Delta^{T}=0.04$
$A_{13}A_{14}$	$A_{14}A_{15}$	$A_{15}A_{16}$	$A_{16}A_{17}$
$A_{13}\left(-126.243,-4309.902\right)$	$A_{14}\left(-134.015,-4516.065\right)$	$A_{15}\left(-141.507,-4706.668\right)$	$A_{16}\left(-148.733,-4882.674\right)$
$A_{14}\left(-134.015,-4516.065\right)$	$A_{15}\left(-141.507,-4706.668\right)$	$A_{16}\left(-148.733,-4882.674\right)$	$A_{17}\left(-155.704,-5044.969\right)$
$T\in \left[-134.015,\;-126.243\right]$	$T\in \left[-141.507,-134.015\right]$	$T\in \left[-148.733,\;-141.507\right]$	$T\in \left[-155.704,\;-148.733\right]$
$T_{E_{13}}=-130.140$	$T_{E_{14}}=-137.770$	$T_{E_{15}}=-145.129$	$T_{E_{16}}=-152.227$
$\Delta^{T}=0.04$	$\Delta^{T}=0.04$	$\Delta^{T}=0.04$	$\Delta^{T}=0.04$
$A_{17}A_{18}$	$A_{18}A_{19}$	$A_{19}A_{20}$	$A_{20}A_{21}$
$A_{17}\left(-155.704,-5044.969\right)$	$A_{18}\left(-162.428,-5194.366\right)$	$A_{19}\left(-168.912,-5331.618\right)$	$A_{20}\left(-175.162,-5457.422\right)$
$A_{18}\left(-162.428,-5194.366\right)$	$A_{19}\left(-168.912,-5331.618\right)$	$A_{20}\left(-175.162,-5457.422\right)$	$A_{21}\left(-181.181,-5572.431\right)$
$T\in \left[-162.428,\;-155.704\right]$	$T\in \left[-168.912,-162.428\right]$	$T\in \left[--175.162,\;-168.912\right]$	$T\in \left[-181.181,\;-175.162\right]$
$T_{E_{17}}=-159.073$	$T_{E_{18}}=-165.677$	$T_{E_{19}}=-172.043$	$T_{E_{20}}=-178.177$
$\Delta^{T}=0.04$	$\Delta^{T}=0.04$	$\Delta^{T}=0.04$	$\Delta^{T}=0.04$
$A_{21}A_{22}$	$A_{22}A_{23}$	$A_{23}A_{24}$	$A_{24}A_{25}$
$A_{21}\left(-181.181,-5572.431\right)$	$A_{22}\left(-186.973,-5677.260\right)$	$A_{23}\left(-192.540,-5772.495\right)$	$A_{24}\left(-197.884,-5858.704\right)$
$A_{22}\left(-186.973,-5677.260\right)$	$A_{23}\left(-192.540,-5772.495\right)$	$A_{24}\left(-197.884,-5858.704\right)$	$A_{25}\equiv B\left(-200,-5891.404\right)$
$T\in \left[-186.973,-181.181\right]$	$T\in \left[-192.540,-186.973\right]$	$T\in \left[-197.884,\;-192.540\right]$	$T\in \left[-200,\;-197.884\right]$
$T_{E_{21}}=-184.083$	$T_{E_{22}}=-189.762$	$T_{E_{23}}=-195.217$	$T_{E_{24}}=-198.943$
$\Delta^{T}=0.04$	$\Delta^{T}=0.04$	$\Delta^{T}=0.04$	$\Delta^{T}=0.00666$

## Data Availability

Data are contained within the article.
